# Nódulo cutáneo de feohifomicosis en una mujer joven

**DOI:** 10.7705/biomedica.7407

**Published:** 2025-08-11

**Authors:** María Carolina Rojas, Karen Viviana Arévalo, Gerzaín Rodríguez

**Affiliations:** 1 Facultad de Medicina, Universidad de La Sabana, Chía, Colombia Universidad de la Sabana Facultad de Medicina Universidad de La Sabana Chía Colombia; 2 Programa de Dermatología, Centro Dermatológico Federico Lleras Acosta E.S.E., Bogotá, D. C., Colombia Programa de Dermatología Centro Dermatológico Federico Lleras Acosta E.S.E Bogotá, D. C. Colombia; 3 Programa de Dermatología, Fundación Universitaria Sanitas, Bogotá, D. C., Colombia Programa de Dermatología Fundación Universitaria Sanitas Bogotá, D. C. Colombia

**Keywords:** feohifomicosis, micosis, dermatología, Phaeohyphomycosis, mycosis, dermatology

## Abstract

Se trata de una mujer de 28 años que, desde hacía cinco años, presentaba un nódulo asintomático de crecimiento lento y de 10 mm de diámetro en el muslo derecho, el cual clínicamente parecía corresponder a un dermatofibroma o a un queloide.

El nódulo se extirpó y la histopatología demostró prominentes granulomas dérmicos ricos en células gigantes, con ocasionales abscesos diminutos y con presencia de abundantes levaduras pigmentadas, de pared oscura, algunas en cadenas, e hifas tabicadas o septadas, de pared negruzca, que inicialmente sugirieron cromoblastomicosis. La abundancia de hifas moniliformes, en cadenas lineales, permitieron concluir que se trataba de una feohifomicosis cutánea, sin penetración a la hipodermis.

Se resaltan la cronicidad de la afección, su forma localizada y la prominencia de granulomas ricos en células gigantes, sin abundancia de abscesos. Se desconoce la evolución de la paciente ya que no regresó a control.

## Abstract (en)

We present a 28 year-old woman with a five-year history of an asymptomatic slowly growing 10 mm nodule on her right thigh, with clinical features suggestive of either a dermatofibroma or a keloid.

The nodule was excised, and histopathological examination revealed prominent dermal granulomas containing numerous giant cells, focal microabscesses, abundant pigmented yeasts with dark walls -some arranged in chains-, and septate hyphae with blackish walls, findings initially suggestive of chromoblastomycosis. The abundance of moniliform hyphae arranged in linear chains allowed us to diagnose cutaneous pheohyphomycosis without hypodermal invasion.

We highlight the chronic nature of the condition, its localized presentation, and the prominence of granulomas rich in giant cells -with scarce abscesses- as notable findings. The patient’s clinical course remains unknown, as she did not return for follow-up.

Se presenta el caso de una mujer de 28 años de edad con una lesión de crecimiento lento y cinco años de evolución en la piel de la cara anterior del muslo derecho, sin antecedentes de trauma ni tratamientos previos. Se apreció un nódulo violáceo de consistencia dura y de 10 mm de diámetro, que sugería un dermatofibroma o un queloide.

El nódulo se extirpó y en el estudio histopatológico se informó epidermis de grosor normal con focos mínimos de paraqueratosis. La epidermis estaba separada de la dermis por una delgada banda de colágeno. Se observó inflamación dérmica difusa con abundantes células gigantes multinucleadas de Langhans, histiocitos y linfocitos, a veces en grupos y frecuentemente con conglomerados diminutos de neutrófilos que formaban microabscesos en el centro de algunos granulomas.

Se apreciaron numerosas levaduras de pared marrón o negruzca, centro claro y superficie irregular; algunas se encontraban fagocitadas y otras libres, en ocasiones dispuestas en cadenas cortas. También, se visualizaron abundantes hifas tabicadas cortas, con pared negra y centro de color ocre o amarillo, usualmente fagocitadas por células gigantes (figuras 1 y 2). La pared pigmentada se demostró mejor con la coloración de Fontana-Masson. El hallazgo se consideró típico de feohifomicosis.

**Figura 1:**
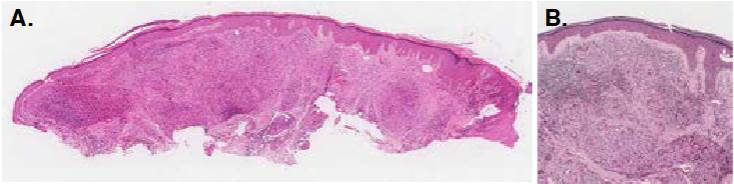
A. Imagen panorámica de la biopsia. Hay acantosis leve y, en la dermis, inflamación granulomatosa difusa. Hematoxilina y eosina, 1X. *B*. Epidermis normal, separada de la dermis por una delgada banda colágena. Granulomas dérmicos particularmente ricos en células gigante multinucleadas de tipo Langhans. Hematoxilina y eosina, 6X.

**Figura 2:**
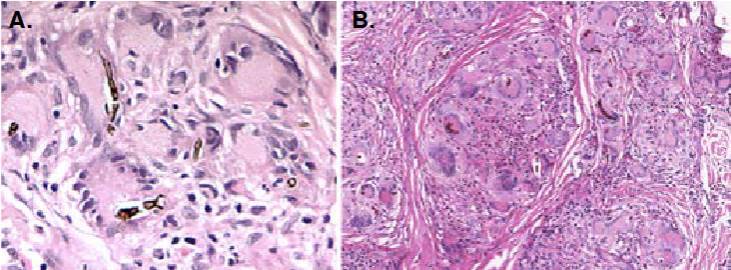
A. Las células gigantes fagocitan levaduras irregulares, gemantes e hifas tabicadas largas, con pigmentación periférica oscura y centro claro. Hematoxilina y eosina, 40X. *B.* Granulomas nítidos ricos en células gigantes que fagocitan levaduras encadenadas e hifas cortas. Hay un absceso pequeño en el granuloma central. Ácido peryódico de Schiff (PAS), 40X.

## Consideraciones éticas

La paciente firmó el consentimiento informado para publicar el caso.

## Discusión

Las feohifomicosis son un grupo de micosis raras producidas por hongos de pared oscura, negra, por lo cual se llaman dematiáceos. Estos hongos comprenden todos aquellos que presentan una coloración oscura o negra en los tejidos o en el cultivo, porque contienen melanina en su pared. El grupo incluye los que producen las feohifomicosis, la cromoblastomicosis y algunos micetomas ^1^^,^^2^. La cromoblastomicosis y los micetomas son excluidos de las feohifomicosis, término creado en 1974 por Ajello *et al.* para establecer esta diferencia ^1^^,^^3^.

La melanina presente en la pared celular de estos hongos les da un aspecto pardo, oscuro o negro, en los tejidos o el cultivo ^1^^,^^2^. Aunque a veces es poco evidente con las coloraciones convencionales, como hematoxilina y eosina o ácido peryódico de Schiff, la melanina se demuestra mejor con las tinciones de Mason-Fontana o plata metenamina de Gomori, como sucedió en este caso. La melanina protege a los hongos de la reacción inmunológica del huésped; les confiere mayor resistencia contra la acción fagocítica de los neutrófilos y los macrófagos ^1^^,^^4^.

Los dematiáceos comprenden más de 60 géneros y cerca de 100 especies. Se identifican por su morfología tisular, por aspectos fisiológicos y mediante métodos moleculares ^2^^,^^5^^-^^7^. Habitan en maderas en descomposición, el suelo, baños caseros, piscinas, aguas estancadas y materia vegetal, y desde allí, pueden ingresar al huésped humano o animal por implantación traumática ^1^^-^^8^. Algunos géneros son: *Bipolaris*, *Alternaria*, *Exophiala*, *Cladophialophora*, *Curvularia*, *Ochronosis*, *Fonsecaea* y *Scedosporium*; las especies de *Alternaria* son los agentes etiológicos más importantes y frecuentes de estas entidades ^1^^-^^7^.

Las feohifomicosis afectan también a animales como ranas, tortugas, peces y gatos ^9^. En el hombre, producen enfermedad cutánea, corneal o diseminada, con compromiso pulmonar, cerebral y meníngeo ^1^^,^^2^^,^^5^ ^8^ ^-^ ^10^.

Las lesiones pueden tener diferente localización, a saber:

- superficiales: capa córnea, cortezas pilosas (tiña negra y piedra negra) y uñas;
- cutánea: en la dermis;
- subcutánea: nódulos de predominio en la hipodermis;
- corneal: queratitis, y
- sistémica o diseminada: con compromiso cutáneo difuso, pulmonar o abscesos cerebrales

En las lesiones superficiales, las levaduras pigmentadas colonizan el estrato córneo y no producen alteraciones visibles. Un hecho para tener en cuenta es que los dematiáceos pueden colonizar lesiones cutáneas producidas por otros hongos y es posible que en el cultivo crezca un hongo dematiáceo y no el que realmente produce la enfermedad ^11^.

La forma cutánea está limitada a la dermis, como en el caso aquí presentado. Puede cursar con pápulas, placas, nódulos y úlceras, lesiones que pueden ser numerosas y generalizadas. Puede ocurrir por colonización de lesiones excoriadas o fisuradas debido al prurito, como en los eccemas plantares ^1^^,^^10^^,^^11^. También, puede ser diseminada.

La forma subcutánea es la más frecuente ^1^^,^^9^^,^^10^. Consiste en un nódulo hipodérmico, circunscrito, encapsulado y de evolución crónica. Se pueden formar abscesos que aumentan su tamaño y simulan un quiste; además, pueden drenar a la superficie cutánea, con notorio daño tisular. El examen histopatológico revela su encapsulación, y los grandes granulomas con abscesos centrales, presencia de hongos pigmentados y, a veces, restos vegetales de la implantación. El compromiso puede diseminarse y generalizarse en individuos inmunodeprimidos ^12^.

La infección de la córnea es rara y origina una queratitis superficial.

La forma sistémica puede ser oportunista y afecta principalmente a personas inmunosuprimidas, diabéticas, con trasplantes o con neoplasias malignas; también, puede inocularse accidentalmente por la inyección cutánea del agente causal durante procedimientos quirúrgicos o por implantación de catéteres contaminados ^1^^,^^9^^,^^10^. Estas condiciones favorecen la gravedad de la enfermedad, llegando a ocasionar compromiso cerebral, pulmonar y cutáneo generalizado, con una tasa de mortalidad hasta del 80 % ^9^^,^^10^^,^^12^^,^^13^.

La histopatología permite hacer el diagnóstico de feohifomicosis, diferenciándolo de muchos otros sugeridos clínicamente, como leishmaniasis, micobacterias atípicas y otras micosis profundas.

En el presente caso, con la forma cutánea, se observó inflamación granulomatosa muy rica en células gigantes, sin abscesos prominentes y centrales en los granulomas, ni hiperplasia. Un aspecto particular de la biopsia de piel fue la ausencia del patrón histológico básico que permite detectar o sospechar micosis profundas, el cual incluye la hiperplasia pseudocarcinomatosa, los abscesos intraepidérmicos y subepidérmicos, y los granulomas con abscesos centrales prominentes. La inexistencia de estos granulomas mixtos, que implica ausencia de neutrófilos, ayuda a explicar la profusión de hifas, pues los polimorfonucleares son una primera línea de defensa con amplia capacidad fagocítica que destruye el hongo ^4^. Es decir, no se ve el patrón típico de las micosis profundas ^11^.

En el tejido examinado, se observaron abundantes levaduras irregulares, pardas o negruzcas, e hifas gruesas, pequeñas, largas, moniliformes, deformadas y toruloideas, es decir, en cadenas unidas entre sí. No se observaron levaduras fumagoides o muriformes, con tabiques horizontales ni verticales, las cuales son propias de los hallazgos propios de la cromoblastomicosis ^14^.

El tratamiento de elección para la feohifomicosis cutánea es la resección de la lesión cuando es localizada ^1^^,^^2^^,^^9^^,^^10^. Los antimicóticos deben usarse preferiblemente combinados. Varios de ellos han demostrado buenos resultados en infecciones fúngicas con especies relacionadas: ketoconazol (400-800 mg/día por dos años), terbinafina (125 mg/día) e itraconazol (100 mg/día) ^15^. Nuevos azoles y equinocandinas, como la micafungina, han producido resultados prometedore, *in vitro* en estudios con aislamientos europeos ^16^.

## Conclusión

La paciente de este reporte presentaba un único nódulo en el muslo derecho, sin factores de riesgo para micosis cutánea. La biopsia evidenció numerosas levaduras e hifas dematiáceas tabicadas dentro de granulomas particularmente ricos en células gigantes fagocíticas y sin abscesos prominentes. La circunscripción de la lesión a la dermis, su crecimiento lento, así como la ausencia de supuración y de senos de drenaje son características de la forma cutánea de la enfermedad. La biopsia por extirpación de la lesión sugirió compromiso de los bordes de la lesión. Sin embargo, la paciente no regresó a control, por lo cual se desconoce su evolución.
